# Using echocardiography to guide the treatment of novel coronavirus pneumonia

**DOI:** 10.1186/s13054-020-02856-z

**Published:** 2020-04-10

**Authors:** Qian-Yi Peng, Xiao-Ting Wang, Li-Na Zhang, Chinese Critical Care Ultrasound Study Group (CCUSG)

**Affiliations:** 1 Department of Critical Care Medicine, National Clinical Research Center for Geriatric Disorders, Xiangya Hospital, Central South University, No. 87 Xiangya Road, Changsha, 410008 Hunan Province China; 2 Department of Critical Care Medicine, Peking Union Medical College Hospital, Peking Union Medical College, Chinese Academy of Medical Sciences, No.1 Shuaifuyuan, Wangfujing, Dongcheng District, Beijing, 100730 China

Up to 24 February 2020, there have been 77,269 officially reported confirmed cases of 2019 novel coronavirus (nCoV) infection in China. Circulatory dysfunction is considered to have a late onset in severe cases of nCoV pneumonia, which is often ignored in clinical treatment. The main causes of acute respiratory failure and subsequent circulatory dysfunction include the rapid progress of lung injury, fluid overload, lung consolidation, and mechanical ventilation for hypoxemia. Most injuries are related to fluid overload, acute lung injury, and long-term hypoxia. Echocardiographic is an important part of critical ultrasonography, which helps to quickly identify the hemodynamic status. We summarized the echocardiographic features of critically ill COVID-19 patients and its clinical use in the treatment of nCoV pneumonia.

## The echocardiographic features of critically ill COVID-19 patients

The echocardiographic features of COVID-19 are mainly related to the severity of disease and cardiovascular complications. Abnormal findings include (1) hyperdynamic cardiac function, presented as the increase of cardiac output (CO) and ejection faction (EF) of the left ventricular (LV), with/without the decrease of peripheral vascular resistance, which is often seen in the early stage following the systemic inflammatory response; (2) acute stress-induced (takotsubo) cardiomyopathy, characterized as LV segmental contraction abnormalities and apical ballooning [1]; (3) right ventricular (RV) enlargement and acute pulmonary hypertension, which are mainly caused by “internal factors” (including alveolar and pulmonary capillary damage caused by inflammation, hypoxia, and hypercapnia, leading to the increase of RV afterload) and “external factors” (including fluid overload, which causes the increase of RV preload, and unsuitable mechanical ventilation parameter setting, which affects the cardiac function by cardiopulmonary interaction); further, LV function will be affected because the right and left hearts are in the same pericardium; and (4) diffuse myocardial inhibition in the late stage, which is often caused by severe hypoxia, and long term of anoxia and inflammation. The echocardiographic features of nCoV pneumonia and their probable causes are shown in Table 1.

**Table 1:** The echocardiographic features of nCoV pneumonia

Features	Echocardiographic manifestations	Causes
Hyperdynamic cardiac function	Increase of cardiac output (CO) and ejection faction (EF) of the left ventricular (LV), with/without the decrease of peripheral vascular resistance	Cardiac stress response to systemic inflammatory response, increase of LV preload by fluid resuscitation, decrease of LV afterload by reduced peripheral vascular resistance.
Acute stress-induced (takotsubo) cardiomyopathy	LV segmental contraction abnormalities and apical ballooning	Elevated levels of circulating plasma catecholamines and its metabolites, microvascular dysfunction, inflammation, estrogen deficiency, spasm of the epicardial coronary vessels, and aborted myocardial infarction.
Right ventricular (RV) enlargement and acute pulmonary hypertension	The end-diastolic area of right ventricular/left ventricular > 0.6. The interventricular septum protruded to the left ventricle, showing the “D-sign.” Decreased systolic and/or diastolic function of RV, changes in frequency and rhythm of pulmonary blood flow, tricuspid valve regurgitation.	The increase in pulmonary vascular resistance caused by hypoxia, pulmonary vasospasm, hypercapnia and inflammation; fluid overload; unsuitable mechanical ventilation parameter setting.
Diffuse myocardial inhibition	Decreased systolic and/or diastolic function of the whole heart.	Severe hypoxia, long term of anoxia and inflammation. The circulatory failure is often caused by diffuse cardiodepression after arrest and the decrease of vascular tension caused by lactic acidosis.

## The protocol of echocardiography examination in nCoV pneumonia

Echocardiography can help to quickly identify the circulatory status of nCoV pneumonia patients and guide hemodynamic management. Five basic views of echocardiography (apical four chamber view, parasternal long axis view, parasternal short axis view, subarachnoid four chamber view, subarachnoid inferior vena cava (IVC) long and short axis view) should be measured, which help to quickly understand the patient’s volume status, cardiac function, and organ perfusion and help to develop hemodynamic management plans. It is suggested to measure the diameter of IVC, EF, velocity-time integral of the left ventricular outflow during continuous and dynamic evaluation of patients’ volume state and fluid responsiveness, left ventricular systolic function, and left ventricular output effect. If necessary, hemodynamic management can follow the “5P” principle, i.e., lower central venous pressure, optimized pulse/heart rate, appropriate pump function and blood pressure, and organ perfusion as the final goal.

## The use of echocardiography in the treatment of nCoV pneumonia

### Fast identify the circulatory status and the types of shock

According to the pathophysiological mechanism of shock, it can be divided into 4 types: distributed shock, cardiogenic shock, hypovolemic shock, and obstructive shock. Critical ultrasonography is of great significance in fast identifying the types of shock and guide hemodynamic management. Since the focused cardiac ultrasound (FOCUS) was proposed in 2010 [2], many different types of FOCUS exams for rapid evaluation of emergency or ICU patients have been introduced, including the focus-assessed transthoracic echocardiography (FATE) advanced FATE protocol [3], fluid administration limited by lung sonography (FALLS) protocol [4], and critical care chest ultrasonic examination (CCUE) protocol [5]. In COVID-19 patients, the most common types of shock are septic shock and cardiogenic shock; however, we still need to exclude obstructive shock (massive pericardial effusion, right heart collapse, heart swing, RV enlargement and “D sign,” tricuspid valve regurgitation, pulmonary artery or deep vein thrombosis, etc.) and hypovolemic shock (decrease of CO, “papillary muscle kissing sign,” IVC collapse and high respiratory variability, etc.) first. Further, we assess whether there are signs supporting cardiogenic shock (enlargement of the heart, segmental or diffuse contraction abnormalities, IVC dilation, B lines in the lungs and pleural effusion, etc.). If the above three kinds of shock are excluded, then we may consider distributed shock according to clinical history and laboratory tests.

### Monitor the right heart function

Novel coronavirus pneumonia may cause the increase in pulmonary vascular resistance due to hypoxia, pulmonary vasospasm, hypercapnia, and inflammation, which further affect the right heart function. Mechanical ventilation itself, especially when lung protective ventilation is not implemented properly, will further increase pulmonary artery pressure and aggravate right heart dysfunction. Right heart dysfunction can be detected by echocardiography, therefore providing important information for circulatory and respiratory management strategies in patients with nCoV pneumonia.

### Monitor the left heart function

Novel coronavirus pneumonia is different from severe acute respiratory syndrome (SARS) in that severe lung injury occurs at the beginning. Some critically ill patients suffer from multiple organ failure, which worsen dramatically in the late stage of disease. It could be a kind of like the “inflammatory storm” with uncontrolled inflammatory reaction in the body. During hypoxia, respiratory distress, intense stress status, and inflammation, the left heart may go through the following abnormalities: segmental dyskinesia, overall hyperdynamic, and diffuse cardiodepression. Diffuse cardiodepression often occurs during lethal hypoxia, in the process of intubation, or after cardiopulmonary resuscitation. The long term of anoxia and inflammation should also be considered. The circulatory failure is often caused by diffuse cardiodepression after arrest and the decrease of vascular tension caused by lactic acidosis. Sepsis or myocardial infarction can also lead to these changes. Left heart function can be evaluated by rapid qualitative and quantitative methods using echocardiography. Critical ultrasonography can also provide etiological evaluation and treatment guidance for patients with systolic dysfunction.

As an important part of critical ultrasonography, echocardiography is a useful tool for the fast screen of circulatory status, identifying the types of shock, monitoring during the respiratory and hemodynamic management, and guiding the treatment of nCoV pneumonia patients, which is especially feasible, convenient, and advantageous in critically ill patients.

## Data Availability

Available.
